# Biological Therapy for Moderate-to-Severe Psoriasis: A 5-Year Analysis of Patients from Lithuania

**DOI:** 10.3390/medicina62050855

**Published:** 2026-04-30

**Authors:** Elada Indrisiunaite, Ieva Renata Jonaityte, Tatjana Karmaziene, Tadas Raudonis

**Affiliations:** 1 Faculty of Medicine, Vilnius University, M. K. Čiurlionio g. 21, 03101 Vilnius, Lithuania; 2 Clinic of Infectious Disease and Dermatovenereology, Institute of Clinical Medicine, Faculty of Medicine, Vilnius University, M. K. Čiurlionio g. 21, 03101 Vilnius, Lithuania

**Keywords:** psoriasis, biological therapy, PASI, DLQI, efficacy, drug survival

## Abstract

*Background and Objectives*: Biological therapy is widely used to treat moderate-to-severe psoriasis. This study aimed to assess the real-world effectiveness and drug survival of biologic treatment in patients with moderate-to-severe psoriasis. *Materials and Methods*: A retrospective study of 210 patients with moderate-to-severe psoriasis who were treated with biological therapy between 2018 and 2023 was conducted. Baseline data included demographics, comorbidities, prior treatments, Psoriasis Area and Severity Index (PASI) and Dermatology Life Quality Index (DLQI) scores, laboratory results, current psoriasis treatment, and treatment-related adverse events. *Results*: Of the 210 patients, 60.0% were male (*n* = 126). The mean age was 48.3 ± 13.6 years in men (range 16–74) and 48.4 ± 13.6 years in women (range 17–79). The mean PASI at initiation of biologic therapy was 15.0 ± 8.1 and decreased to 3.3 ± 4.7 at 1 year, 2.7 ± 4.0 at 3 years, and 2.8 ± 3.3 at 5 years. Drug discontinuation differed between therapies: etanercept had a higher hazard of discontinuation than ustekinumab (hazard ratio (HR) 2.55, 95% confidence interval (CI) 1.17–5.52; *p* = 0.0179), infliximab (HR 0.36, 95% CI 0.13–0.97; *p* = 0.0429) and adalimumab (HR 0.47, 95% CI 0.23–0.98; *p* = 0.0453). *Conclusions*: In routine clinical practice, biologic therapy was associated with substantial and sustained improvements in the PASI over up to 5 years of follow-up. Drug survival was initially high for all agents but separated over time, with etanercept showing the poorest long-term persistence and a higher hazard of discontinuation compared with other drugs.

## 1. Introduction

Psoriasis is a chronic, immune-mediated systemic disease affecting more than 125 million people worldwide [1,2,3,4]. In Lithuania specifically, approximately 76,000 patients are estimated to carry a diagnosis of psoriasis, with a prevalence of approximately 2% [5]. As a lifelong condition, patients with moderate-to-severe disease often require long-term systemic therapy to maintain stable disease control. Although psoriasis is typically manageable rather than curable, modern treatment strategies can achieve sustained symptom control over prolonged periods.

Disease severity and treatment response are usually evaluated using the Psoriasis Area and Severity Index (PASI) [6]. Achieving a ≥75% reduction from the baseline (PASI 75) in PASI scores after treatment has been the main therapeutic goal for a long time, but with the advent of biologics, a ≥90% reduction from baseline (PASI 90) or even a ≥100% reduction from baseline (PASI 100) have become realistic targets within routine care for many patients with moderate-to-severe disease [7]. Patient-reported quality of life is simultaneously assessed using the Dermatology Life Quality Index (DLQI), a validated 10-item questionnaire that captures the broader impact of psoriasis on daily functioning and is increasingly used alongside PASI as a co-primary outcome measure in both clinical trials and real-world studies [8,9,10].

Treatment selection is guided by disease severity. Mild psoriasis is managed mainly with topical therapies (e.g., corticosteroids, vitamin D analogues, calcineurin inhibitors, tazarotene, coal tar, anthralin) [11]. Moderate-to-severe psoriasis often requires phototherapy, conventional systemic agents (e.g., methotrexate, cyclosporine, acitretin, fumaric acid esters), small-molecule therapies (e.g., apremilast), or biologic therapies. Biologic therapy is typically used when conventional systemic treatment is ineffective, not tolerated, or contraindicated [7,12].

Current biologic options include tumour necrosis factor (TNF) inhibitors (infliximab, adalimumab, etanercept), interleukin (IL)-12/23 inhibitor (ustekinumab), IL-17 inhibitors (secukinumab, ixekizumab, bimekizumab, brodalumab), and IL-23 inhibitors (guselkumab, risankizumab). However, no single biologic is optimal for all patients because mechanisms of action, efficacy, and adverse event profiles differ across agents [13]. While biologic therapy is primarily evaluated in adult populations, several agents—including Ustekinumab—have received regulatory approval for use in adolescent patients aged 6 years and older, with approved weight-based dosing regimens for this age group [14,15]. Nevertheless, real-world data on the effectiveness and safety of biologic therapy specifically in adolescent patients with moderate-to-severe psoriasis remain limited.

Real-world studies show that biologics can deliver strong effectiveness and patient acceptability, often outperforming conventional systemic agents in both clinical and patient-reported outcomes [16,17,18,19]. EuroGuiDerm guidelines recommend systemic therapy (conventional or biologic) for moderate to severe psoriasis, but access and implementation vary across Europe—particularly in Central and Eastern Europe—largely due to national guidelines and reimbursement rules [20,21]. In the Baltic countries, the number of patients per 100,000 inhabitants receiving biologic therapy remains considerably lower than in Western Europe, reflecting suboptimal access due to multiple factors including reimbursement conditions and availability of treatments [22].

Long-term drug survival—defined as the time a patient continues on a given biologic without discontinuation—is an important real-world measure of sustained treatment effectiveness and tolerability. Understanding the factors associated with biologic discontinuation and switching patterns is essential for optimising treatment sequencing and improving long-term patient outcomes in clinical practice. The choice of biologic therapy is also strongly influenced by comorbidities. Psoriasis is closely associated with several comorbid conditions, particularly psoriatic arthritis (PsA), cardiometabolic diseases (obesity, hypertension, diabetes, and dyslipidaemia), gastrointestinal disease (inflammatory bowel disease, especially Crohn’s disease), psychosocial disorders, infections, and malignancies [2]. These comorbidities complicate biologic selection in routine practice and can further impair patients’ quality of life [23].

Despite the growing body of real-world evidence from large Western European and North American registries, data from Central and Eastern European countries—including Lithuania—remain scarce. National prescribing regulations, reimbursement criteria, and patient population characteristics in this region may differ substantially from those reported in international registries, limiting the direct generalisability of existing findings to Lithuanian clinical practice.

To address these real-world decision needs, we report five years of outcomes from a Lithuanian tertiary dermatovenereology centre, evaluating effectiveness (PASI, DLQI scores), drug survival and switching patterns, and safety across TNF, IL-12/23, IL-17, and IL-23 biologic therapies.

## 2. Materials and Methods

A retrospective medical record review was conducted for 210 patients who received biologic therapy between 2018 and 2023 at Vilnius University Hospital Santaros Klinikos, Centre of Dermatovenereology (VUH SK DVC). As one of two centres in Lithuania authorised to initiate biological therapy for moderate-to-severe psoriasis, VUH SK DVC receives referrals from across the country. However, as a tertiary referral centre, the cohort is likely to include patients with more severe, complex, or refractory disease than those seen in primary or secondary care settings, and the findings may not be fully generalisable to the broader psoriasis population in Lithuania or beyond. The study protocol was approved by the Regional Biomedical Research Ethics Committee (ethical approval number 2025/7-1683-1132, approval date: 8 July 2025). In total, 210 patients aged 16–79 years were included. Biologic agents targeting tumour necrosis factor-α or interleukins 12/23, 23, and 17 were prescribed after a diagnosis of moderate-to-severe plaque psoriasis (PASI and/or DLQI ≥ 10) had been established, when the disease had persisted for more than 6 months, and when topical therapies, photo (chemo)therapy, and at least one conventional systemic agent had been insufficiently effective or not tolerated.

Baseline characteristics included comorbidities, demographic data, prior therapies, DLQI score, PASI score, laboratory test results, current psoriasis treatment, and treatment-related adverse events. Statistical analysis was performed using Microsoft Excel (version 16.0) and MATLAB R2025b (version 25.2; MathWorks, Natick, MA, USA). A *p*-value < 0.05 was considered statistically significant.

## 3. Results

### 3.1. Baseline Demographic and Other Clinical Characteristics

Among the 210 patients included in this study, 60.0% (*n* = 126) were male. The mean age of male patients was 48.3 ± 13.6 years (range 16–74 years), and that of female patients was 48.4 ± 13.6 years (range 17–79 years). A positive family history of psoriasis was documented in 28.6% of patients. The mean body mass index (BMI) at the start of biologic therapy was 30.0 ± 7.0 kg/m^2^. The mean duration of psoriasis was 24.5 ± 12.0 years. The most frequently affected anatomical sites were the legs (91.0%), arms (88.6%), scalp (78.1%), and torso (70.0%). Psoriatic onychodystrophy was observed in 83.8% of patients (*n* = 176) and psoriatic arthropathy in 52.9% (*n* = 111).

Patients were treated with four classes of biologic agents: TNF-α inhibitors (adalimumab, etanercept, infliximab), the IL-12/23 inhibitor ustekinumab, IL-17 inhibitors (secukinumab, ixekizumab) and IL-23 inhibitors (risankizumab, guselkumab). Overall, 40.0% (*n* = 84) received adalimumab, 21.9% (*n* = 46) ustekinumab, 11.9% (*n* = 25) guselkumab, 11.4% (*n* = 24) risankizumab, 5.7% (*n* = 12) infliximab, 4.8% (*n* = 10) etanercept, 3.3% (*n* = 7) secukinumab and 1.0% (*n* = 2) ixekizumab. Because the ixekizumab group was very small, these patients were excluded from subsequent statistical analyses.

Biologic agents were administered according to their approved dosing schedules. Adalimumab was initiated at 80 mg subcutaneously, followed by 40 mg at week 1 and then 40 mg every 2 weeks. Etanercept was given subcutaneously at 25–50 mg weekly or twice weekly. Infliximab was administered intravenously at 5 mg/kg at weeks 0, 2, and 6, then every 8 weeks. Ustekinumab was given subcutaneously at 45 mg (or 90 mg for patients >100 kg) at weeks 0 and 4, then every 12 weeks. In the adolescent patients included in the cohort (*n* = 2), ustekinumab was dosed according to the approved weight-based regimen for patients aged 6 to 17 years: 0.75 mg/kg for those weighing less than 60 kg, 45 mg for those weighing between 60 and 100 kg, and 90 mg for those weighing more than 100 kg, administered on the same schedule. Due to the very small number of adolescent patients, a dedicated subgroup analysis could not be performed.

Secukinumab was administered at 300 mg weekly for 5 weeks, then every 4 weeks. Risankizumab was given at 150 mg at weeks 0 and 4, then every 12 weeks, and guselkumab at 100 mg at weeks 0 and 4, then every 8 weeks.

Prior phototherapy had been administered in 69.1% of patients (*n* = 145). Methotrexate was used before initiation of biologic therapy by 88.1% (*n* = 185), whereas 30.5% (*n* = 64) received methotrexate concomitantly with biologic therapy. Concomitant topical treatment was highly prevalent: 97.6% (*n* = 205) used emollients, 94.8% (*n* = 199) topical corticosteroids, and 48.6% (*n* = 102) keratolytic agents.

Over the entire observation period, the biologic agent was changed a mean of 1.2 ± 1.4 times per patient, and 53 patients required at least one switch during the 5-year study period. The overall mean duration of biologic therapy was 147.2 ± 87.9 weeks, with no clinically meaningful differences in treatment duration across individual agents. All baseline demographic and clinical characteristics of patients are presented in Table 1.

### 3.2. Baseline PASI and Changes During Treatment

**Figure 1:**
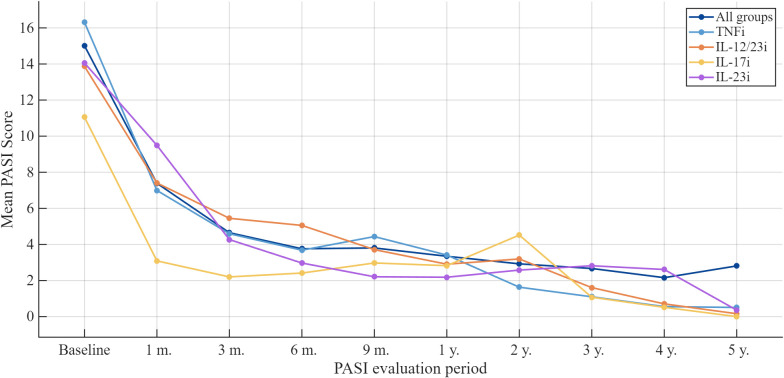
Mean PASI scores of all patients during biologic therapy.

The overall mean PASI at the initiation of biologic therapy was 15.0 ± 8.1, with baseline values differing across treatment groups: 14.7 ± 6.9 for adalimumab, 15.2 ± 9.1 for guselkumab, 12.8 ± 6.4 for risankizumab, and 13.9 ± 8.3 for ustekinumab. After one year of treatment, the mean PASI score decreased to 3.3 ± 4.7, further declining to 2.7 ± 4.0 after three years and to 2.8 ± 3.3 after five years. These findings demonstrate a sustained and progressive improvement in the PASI over the 5-year follow-up period (Figure 1).

#### 3.2.1. Overall Efficiency

A covariate-adjusted linear mixed-effects model with random patient intercepts (460 observations) was used to evaluate the PASI over time by biologic treatment, adjusting for sex, age, disease duration, baseline PASI values, BMI, concomitant methotrexate use, psoriatic arthropathy, and psoriatic onychodystrophy (PASI ~ 1 + Sex + Age + DiseaseDuration + PASI_BL + BMI + MTX + Arthropathy + Onychodystrophy + Drug × Visit + (1|PatientID), REML), with adalimumab and baseline as reference levels.

After adjustment for baseline covariates, a significant main effect of drug was observed (F (3, 415) = 4.90, *p* = 0.002), indicating that PASI scores differed significantly between biologics, as averaged across visits. The interaction of drug and visit remained statistically significant (F (24, 415) = 1.79, *p* = 0.013), confirming that PASI trajectories over time differed between drugs. None of the included baseline covariates—sex, age, disease duration, baseline PASI values, BMI, concomitant methotrexate use, psoriatic arthropathy, or onychodystrophy—reached statistical significance as independent predictors of PASI score in the adjusted model (all *p* > 0.05). However, it should be noted that non-significance does not imply equivalence or the absence of residual confounding; the possibility that unmeasured or incompletely adjusted baseline differences contributed to the observed treatment trajectories cannot be excluded and should be considered when interpreting these findings.

#### 3.2.2. Efficacy: PASI 50 Response

**Figure 2:**
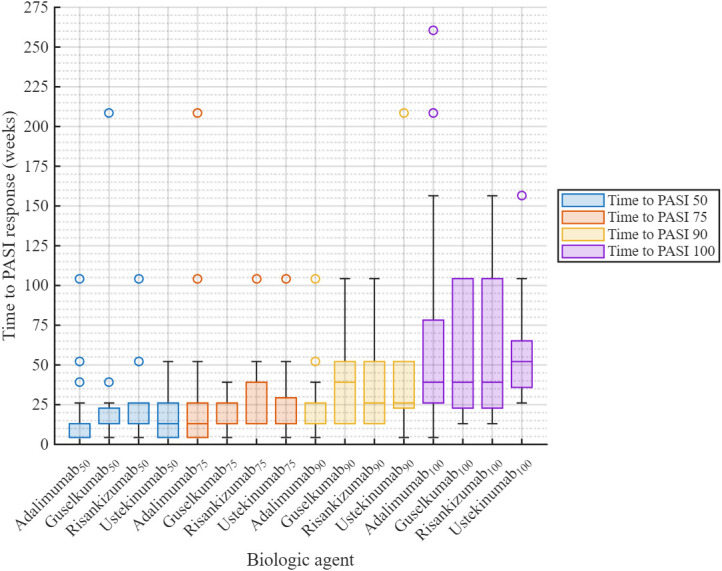
Time to achieve PASI 50, PASI 75, PASI 90, and PASI 100, by biologic agent. PASI 50, 75, 90, and 100 indicate ≥50%, ≥75%, ≥90%, and 100% improvement from baseline, respectively.

Across all biologic agents, high rates of at least a 50% reduction from baseline in the Psoriasis Area and Severity Index score (PASI 50) were observed. The proportion of patients achieving PASI 50 at least once during follow-up was highest with risankizumab and infliximab (100.0% each), followed by guselkumab (92.0%, *n* = 23), etanercept (90.0%, *n* = 9), ustekinumab (87.0%, *n* = 40), secukinumab (85.7%, *n* = 6), and adalimumab (83.3%, *n* = 70). No statistically significant difference in the time to achieve PASI 50 was observed among treatment groups (*p* = 0.317); however, this finding should be interpreted with caution given the small sample sizes in several subgroups and does not allow conclusions regarding comparable efficacy between agents (Figure 2). A minority of patients in each group exhibited prolonged times to PASI 50 response (>50 weeks, with isolated cases exceeding 100–200 weeks), reflecting inter-individual variability in treatment response.

#### 3.2.3. Efficacy: PASI 75 Response

The time to first achieve a 75% reduction from baseline in the Psoriasis Area and Severity Index score (PASI 75) was consistently short across all four biologic therapies, with median values remaining well below 50 weeks. For adalimumab, guselkumab, and ustekinumab, the time to first PASI 75 was 13.0 weeks. Guselkumab showed a relatively tight interquartile range, suggesting more consistent response times, whereas risankizumab exhibited the widest interquartile range, reflecting greater inter-individual variability. No statistically significant differences were observed in pairwise comparisons (adalimumab vs. guselkumab: *p* = 0.967; adalimumab vs. risankizumab: *p* = 0.820; adalimumab vs. ustekinumab: *p* = 0.796; guselkumab vs. risankizumab: *p* = 0.703; guselkumab vs. ustekinumab: *p* = 0.683; risankizumab vs. ustekinumab: *p* = 0.999), and the overall median time to PASI 75 did not differ significantly among groups (*p* = 0.573); however, the absence of statistically significant differences does not imply equivalent efficacy between agents, particularly given the limited statistical power resulting from small subgroup sizes. The distribution was right-skewed, with a small number of outliers showing a markedly prolonged time to PASI 75 (Figure 2).

**Figure 3:**
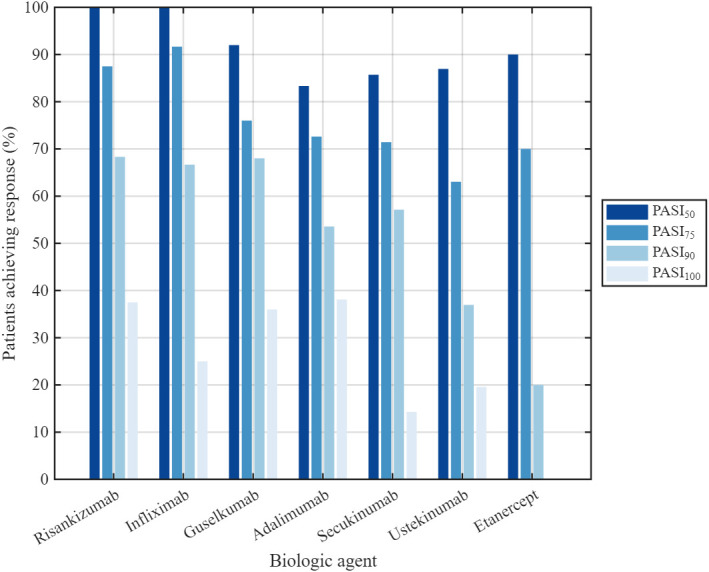
Proportion of patients achieving PASI 50, PASI 75, PASI 90 and PASI 100 responses.

High PASI 75 response rates were observed with infliximab (91.7%; *n* = 11) and risankizumab (87.5%; *n* = 21), with patients achieving PASI 75 at least once during follow-up. A smaller proportion of patients achieved PASI 75 with guselkumab (76.0%, *n* = 19), adalimumab (72.6%, *n* = 61), and secukinumab (71.4%, *n* = 5). The lowest PASI 75 response rate was recorded in the etanercept group (70.0%, *n* = 7) (Figure 3).

#### 3.2.4. Efficacy: PASI 90 Response

As anticipated for a more stringent response threshold, the time to achieve a 90% reduction from baseline in the Psoriasis Area and Severity Index score (PASI 90) was both longer and more variable across treatment groups compared with the time to PASI 75. The median time was 26.1 weeks for adalimumab, risankizumab, and ustekinumab, and 39.1 weeks for guselkumab. Variability was greater for guselkumab and risankizumab (wider interquartile ranges) than for adalimumab and ustekinumab. No statistically significant differences were observed in the overall comparison (*p* = 0.226) or in any pairwise analysis (all *p* ≥ 0.372); however, these results should be interpreted descriptively given the limited sample sizes in several subgroups, and they do not allow for definitive conclusions regarding comparative efficacy between agents. A small number of outliers with time extensions exceeding 100–200 weeks were noted (Figure 2).

The highest PASI 90 response rates were observed with guselkumab (68.0%; *n* = 17) and infliximab (66.7%; *n* = 8), followed by risankizumab (62.5%, *n* = 15), secukinumab (57.1%, *n* = 4), and adalimumab (53.6%, *n* = 45). The lowest rate was with etanercept (20.0%, *n* = 2) (Figure 3).

#### 3.2.5. Efficacy: PASI 100 Response

The time to achieve complete skin clearance (PASI 100) was the longest and most variable of all PASI endpoints. The median time to PASI 100 was 39.1 weeks in the adalimumab, guselkumab, and risankizumab groups and 52.1 weeks in the ustekinumab group. Despite this numerical difference, no statistically significant differences were detected in pairwise comparisons (all *p* ≥ 0.969) or in the overall group comparison (*p* = 0.977); these results should be interpreted descriptively, as the small sample sizes in several biologic subgroups substantially limit statistical power and preclude definitive comparative conclusions between agents. Isolated outliers exceeding 200 weeks were observed, most notably in the adalimumab group (Figure 2).

PASI 100 response rates were highest with adalimumab (38.1%; *n* = 32) and risankizumab (37.5%; *n* = 9), followed by guselkumab (36.0%; *n* = 9). A smaller proportion of patients achieved complete clearance in the infliximab (25.0%, *n* = 3) and secukinumab (14.3%, *n* = 1) groups, whereas no patients in the etanercept group achieved PASI 100 (Figure 3).

### 3.3. Baseline DLQI and Its Changes During Treatment

**Figure 4:**
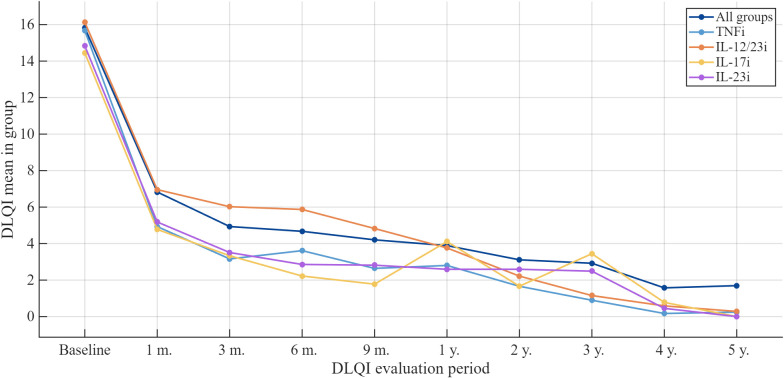
Mean Dermatology Life Quality Index (DLQI) scores over time across all biologic treatments.

At baseline, the mean DLQI in our study was 15.8 ± 7.2 (*n* = 206). Mean DLQI values were then calculated at multiple time points during follow-up: 6.8 ± 5.9 at 1 month (*n* = 167), 4.9 ± 5.8 at 3 months (*n* = 165), 4.7 ± 6.0 at 6 months (*n* = 174), 4.2 ± 5.2 at 9 months (*n* = 156), 3.9 ± 5.7 at 1 year (*n* = 163), 3.1 ± 5.0 at 2 years (*n* = 135), 2.9 ± 5.6 at 3 years (*n* = 103), 1.6 ± 2.5 at 4 years (*n* = 47) and 1.7 ± 3.0 at 5 years (*n* = 23), indicating a rapid improvement during the first months and a gradual further decline thereafter (Figure 4).

**Figure 5:**
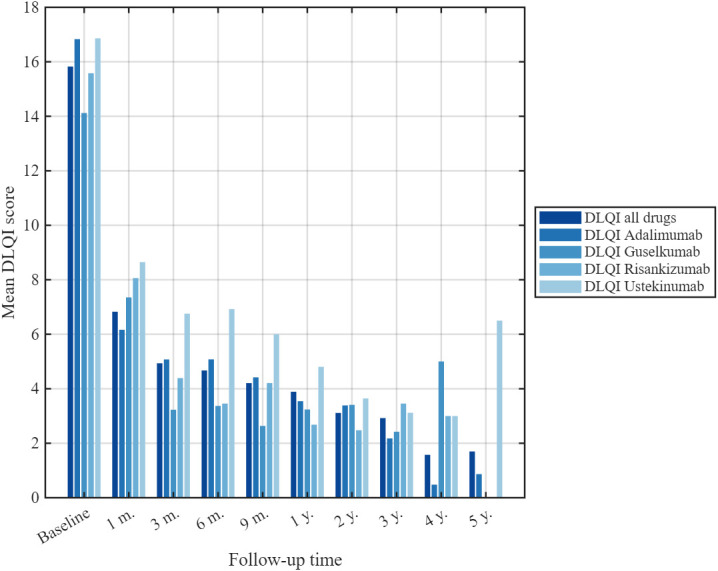
DLQI scores over time for each biologic treatment.

Baseline and 1-year DLQI scores by biologic agent were as follows: ustekinumab 16.7 ± 7.8 and 4.8 ± 5.5; risankizumab 15.6 ± 6.2 and 2.7 ± 3.9; guselkumab 14.1 ± 6.5 and 3.2 ± 4.5; adalimumab 16.8 ± 6.9 and 3.5 ± 5.8. Thus, adalimumab produced the largest mean reduction in the DLQI over the first year, whereas risankizumab had the lowest absolute DLQI score at 12 months, indicating the smallest residual impact of psoriasis on patients’ quality of life. Mean changes in the DLQI across all treatment groups are presented in Figure 5.

### 3.4. Switching of Biologics

Over the 5-year follow-up, 154 patients remained on their initial biologic therapy without switching. Among the 53 patients who required at least one switch, insufficient efficacy was the primary reason (93.8%). Safety-related switches were uncommon and included: multiple non-melanoma skin tumours and adalimumab-induced paradoxical psoriasis (two separate adalimumab-treated patients), gastroesophageal reflux disease, and a case of blurred vision with injection-site reactions.

**Figure 6:**
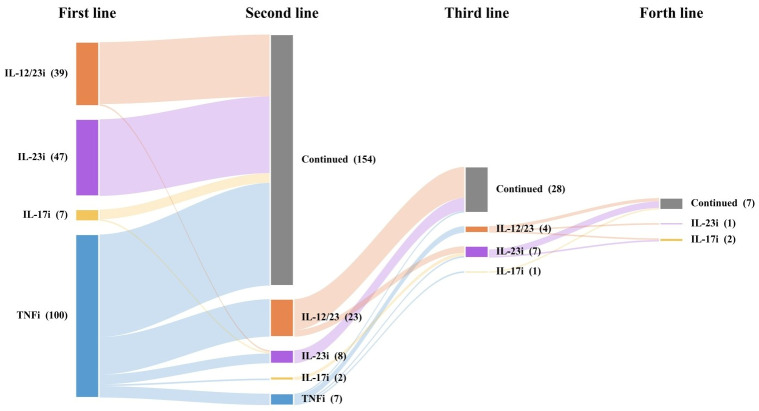
Frequency of biologics switching and switching patterns.

Among patients who switched, the first-line agent was adalimumab in 50.0% (*n* = 20), etanercept in 22.5% (*n* = 9), infliximab in 17.5% (*n* = 7), and ustekinumab in 7.5% (*n* = 3), with adalimumab-to-ustekinumab being the most frequent pattern (35.7%, *n* = 20). Following the switch, PASI 75 was achieved in 60.0%, 61.5%, and 100% of patients undergoing their second, third, and fourth biologic, respectively, within a mean of 27.2, 13.0, and 19.6 weeks. The switching patterns are illustrated in Figure 6.

### 3.5. Additional Topical Treatment and Phototherapy

Topical agents were prescribed to all patients during biological therapy. Emollients were used by 99.0% (*n* = 206) of patients, and topical glucocorticoids by 94.8% (*n* = 199). Keratolytic preparations were prescribed in 48.6% of cases (*n* = 102), whereas topical calcineurin inhibitors were used much less frequently (11.4%; *n* = 24). In addition, 69.1% of patients (*n* = 145) received narrowband 311 nm ultraviolet B phototherapy (UVB); among them, 21.9% (*n* = 46) underwent whole-body and 18.1% (*n* = 38) local phototherapy. The distribution of concomitant topical treatments use across biologic cohorts is presented in Table 2.

### 3.6. The Use of Methotrexate Before and During Biological Therapy

Before starting biologic treatment, methotrexate had been prescribed to 88.1% of patients (*n* = 185). In most cases (91.9%, *n* = 170), the dose remained unchanged, while dose reduction and escalation were relatively uncommon, occurring in 4.3% (*n* = 8) and 3.8% (*n* = 7), respectively. The most frequently reported adverse effects attributed to methotrexate were nausea (20.0%, *n* = 37) and general weakness (14.1%, *n* = 26). Less common adverse events included elevated liver enzyme levels (8.1%, *n* = 15), severe headache (4.9%, *n* = 9), vomiting (3.2%, *n* = 6), abdominal discomfort (2.7%, *n* = 5), diarrhoea (2.7%, *n* = 5), dizziness (2.7%, *n* = 5), stomach pain (2.2%, *n* = 4) and recurrent upper respiratory tract infections (1.1%, *n* = 2).

During biologic therapy, concomitant methotrexate was used in 30.5% of patients (*n* = 64), with the highest rate in the adalimumab group (35.7%, *n* = 30). Among these, 21.9% (*n* = 14) subsequently discontinued methotrexate, most commonly due to psoriasis regression (28.6%, *n* = 4) and general weakness (28.6%, *n* = 4). The methotrexate dose was reduced in 20.3% (*n* = 13), increased in 4.7% (*n* = 3), and left unchanged in 75.0% (*n* = 48) of patients on combination treatment. The distribution of concomitant methotrexate use across biologic cohorts is presented in Table 2.

### 3.7. Comorbidities

Dermatological comorbidities accounted for 32.3% of all recorded conditions. The most prevalent were infectious skin diseases (predominantly fungal infections of the skin and nails, 12.4%), inflammatory skin diseases (seborrheic dermatitis, 9.5%), and skin cancer and precancerous lesions (basal cell carcinoma, 8.3%). Non-dermatological comorbidities represented 67.7% of the total. The most frequent were cardiometabolic and cardiovascular conditions, led by hypertension (17.5%, *n* = 62), followed by dyslipidaemia (7.3%), and obesity (7.1%). Infectious diseases were the second most common non-dermatological category (19.8%), of which coronavirus disease (COVID-19) was the most frequently recorded (4.8%, *n* = 17). All comorbidity groups are presented in detail in Table 3.

### 3.8. Drug Survival Rates of Biological Agents

**Figure 7:**
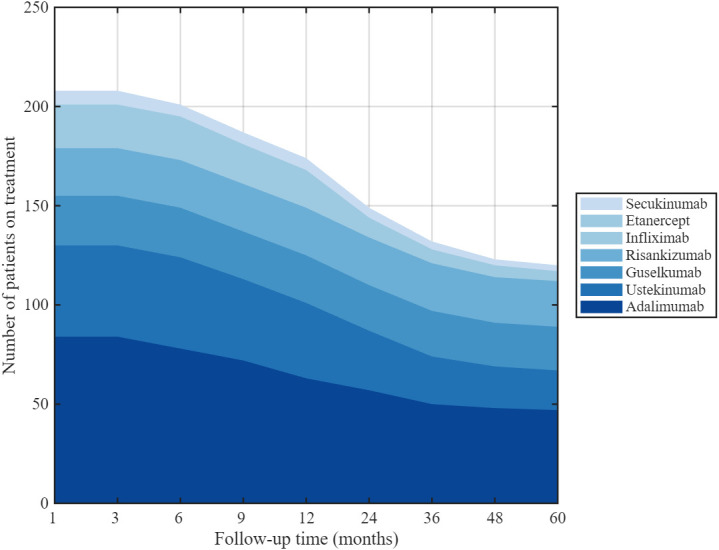
Number of patients treated with each biologic agent.

Drug survival was initially high across all agents and declined gradually over follow-up (Figure 7). At 1 year, survival ranged from 75.0% (adalimumab) to 100% (risankizumab), with intermediate values of 82.6% (ustekinumab), 96.0% (guselkumab), 91.7% (infliximab), 85.7% (secukinumab), and 80.0% (etanercept). By 5 years, a marked separation between agents was evident: risankizumab maintained the highest survival (95.8%), followed by guselkumab (88.0%), adalimumab (56.0%), secukinumab (42.9%), ustekinumab (43.5%), infliximab (33.3%), and etanercept (10.0%). Figure 7 shows the number of patients treated with each biologic agent.

**Figure 8:**
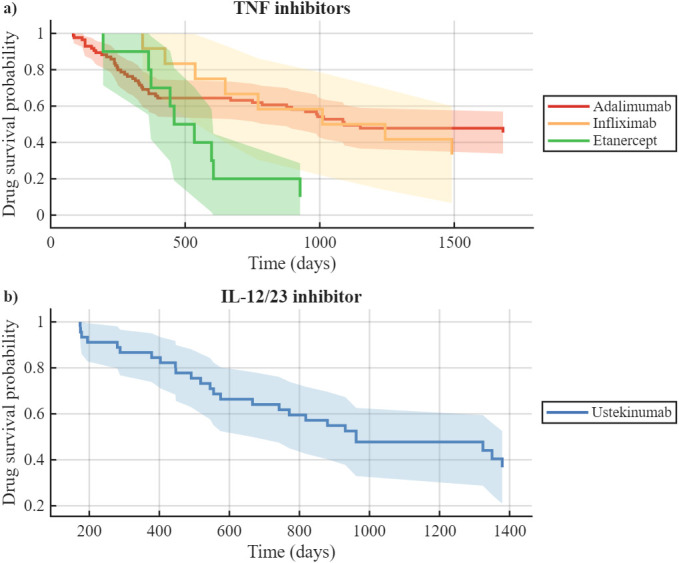
Kaplan–Meier drug survival curves grouped by biologic classes for all patients: (**a**) TNF inhibitors; (**b**) IL-12/23 inhibitors.

The unadjusted Cox proportional hazards model indicated that etanercept was associated with a significantly higher hazard of discontinuation compared with ustekinumab (HR 2.55, 95% CI 1.17–5.52; *p* = 0.018), infliximab (HR 0.36, 95% CI 0.13–0.97; *p* = 0.043), and adalimumab (HR 0.47, 95% CI 0.23–0.98; *p* = 0.045). No significant differences in discontinuation risk were observed among adalimumab, infliximab, and ustekinumab (all *p* > 0.79) (Table 4). The proportional hazards assumption held for all pairwise models except adalimumab vs. etanercept (Figure 8).

## 4. Discussion

### 4.1. Efficacy

A 2023 meta-analysis reported that infliximab, IL-17, and IL-23 inhibitors (except tildrakizumab) were significantly more likely to achieve PASI 90 than ustekinumab, the other TNF-α inhibitors, and deucravacitinib. In addition, adalimumab, tildrakizumab, and ustekinumab showed better results than etanercept [7].

Another review, published in the United States (US) in 2021, noted that newer anti-IL-17 and anti-IL-23 agents (brodalumab, ixekizumab, risankizumab) generally demonstrate higher response rates than anti-TNF-α therapies and ustekinumab across multiple endpoints (PASI 75/90/100) and across short- and longer-term follow-up [24].

A 2020 study also supports the superior efficacy of secukinumab over ustekinumab for high-level responses: at week 52, secukinumab was superior in the proportion achieving PASI 90. This trial also reported sustained benefits in skin clearance and quality of life through 52 weeks, with a safety profile consistent with prior studies [25]. Secukinumab also showed a more rapid onset, with significantly more patients achieving responses by week 4 compared with ustekinumab. Although ustekinumab is effective in moderate to severe plaque psoriasis, 40–50% did not achieve PASI 90 [25,26,27,28].

Other pivotal trials similarly demonstrate strong efficacy for newer agents: over 12 weeks, both ixekizumab dosing regimens were more effective than placebo and etanercept [29]. Risankizumab also demonstrated superior efficacy compared with placebo and ustekinumab in moderate-to-severe plaque psoriasis, with similar treatment-emergent adverse event profiles across groups [30]. Finally, long-term maintenance data for infliximab indicate that while ~80% achieved PASI 75 at week 10, 61% maintained PASI 75 at week 50 with 5 mg/kg every 8 weeks [31,32,33].

In our study, for PASI 75, the median time to response was 13.0 weeks for adalimumab, guselkumab, and ustekinumab, indicating similar response rates with substantial inter-individual variability. PASI 75 rates ranged from 70.0 to 91.7%, the highest with infliximab (91.7%) and the lowest with etanercept (70.0%). For PASI 90, median times were 26.1 weeks for adalimumab, risankizumab, and ustekinumab, and 39.1 weeks for guselkumab; response kinetics were broadly comparable overall, with no statistically significant between-group differences. PASI 90 rates ranged from 20.0% to 68.0%, with the highest rate observed with guselkumab (68.0%) and the lowest with etanercept (20.0%). For PASI 100, the median time was 39.1 weeks for adalimumab, guselkumab, and risankizumab, and 52.1 weeks for ustekinumab, suggesting a later median time to complete clearance with ustekinumab. PASI 100 rates were generally low (0–38.1%); the highest was with adalimumab (38.1%) and absent with etanercept (0%).

Our efficacy results are broadly consistent with data from large European real-world registries. The Danish national DERMBIO registry found smoking and increased body weight to be independent negative predictors of PASI ≤ 2 at 6 months across all biologic classes [34]; the long disease duration (median 24.5 years) and complex patient profiles of our tertiary referral cohort may partly explain the response patterns observed, although direct comparisons with registry data should be made with caution. The British Association of Dermatologists Biologics and Immunomodulators Register (BADBIR) data also support the use of PASI 90 and PASI 100 as primary treatment targets, confirming that an absolute PASI ≤ 2 is the most clinically relevant endpoint in routine practice [35]. The sustained reduction in the PASI over five years recorded in our study is also consistent with DERMBIO evidence linking early deep response with more stable long-term disease control and improved survival after medication [36]. Overall, the efficacy results from our Lithuanian cohort appear broadly aligned with patterns reported in European real-world settings, although direct comparisons are limited by the single-centre nature of the present study, the small sizes of several biologic subgroups, and potential differences in patient selection, prescribing practices, and follow-up duration between settings. These findings should therefore be interpreted as preliminary and hypothesis-generating rather than conclusive.

### 4.2. DLQI

The Dermatology Life Quality Index (DLQI) is a 10-item questionnaire that assesses how skin disease affects health-related quality of life across six domains: symptoms and feelings, daily activities, leisure, work/school, personal relationships, and treatment. Scores range from 0 to 30, with higher scores indicating worse quality of life; a score of 0 indicates no impact, whereas 30 indicates an extremely large impact on the patient’s life [8]. Since 2018, the DLQI has been mandatory for assessment in Lithuania. Under Lithuanian reimbursement rules, if after 3 months PASI improvement is 50–74% and DLQI ≤ 5, treatment is continued; if DLQI > 5, treatment should be changed [37].

Multiple studies show that better skin clearance is associated with clinically meaningful improvements in DLQI. Mattei et al. reported that a 45–55% mean PASI improvement was associated with an average 5-point DLQI improvement (*p* < 0.01), while an ~85% mean PASI improvement corresponded to an average 10-point DLQI improvement [9]. Puig et al. reported similar results: with adalimumab or infliximab, a 75–89% PASI improvement was associated with mean DLQI improvements of 8.5 (*p* < 0.01) and 8.67 (*p* not reported), respectively. When PASI improvement was ≥90%, mean DLQI improvements were 10.7 (*p* < 0.01) and 8.95 (*p* not reported), respectively [38]. In patients treated with ustekinumab, achieving PASI 75 has been associated with substantial quality-of-life benefits, with a mean improvement in DLQI of 12.57 points at week 24. This supports the broader conclusion that greater PASI responses are linked to better DLQI outcomes [39,40].

Comparative studies also suggest that biologics may differ in their impact on quality of life. Strober et al. reported greater improvements in DLQI with ustekinumab than with adalimumab or etanercept at 6 and 12 months, although key potential confounders (such as dose adjustments and concomitant systemic therapy) were not controlled [41]. In the CLARITY trial, secukinumab led to faster and more sustained improvements in DLQI than ustekinumab [25]. Finally, registry data suggest that patients treated with etanercept may be less likely to achieve minimal DLQI impairment (DLQI 0/1) than those treated with adalimumab. This aligns with reports of higher etanercept discontinuation rates due to insufficient effectiveness [42].

The rapid and sustained improvement in the DLQI observed in our cohort appears broadly aligned with patterns reported in large international real-world studies, although direct comparisons are limited by differences in study design, patient selection, and sample size. Data from the Danish DERMBIO registry showed that biologic therapy is associated with significant improvements in patient-reported quality of life, with DLQI significantly decreasing during the first months of treatment and continuing to improve over longer follow-up periods, mirroring the trajectory observed in our cohort [10]. Loft et al. reported that symptoms and feelings were the largest contributors to quality-of-life decline at baseline, and PASI changes were moderately correlated with DLQI changes over the first 12 months of treatment, a finding consistent with our observation that DLQI improvements closely followed PASI decreases across all biologic classes [10]. Similarly, data from the PSOLAR registry confirmed that greater skin clearance is associated with clinically meaningful improvements in DLQI, with patients achieving PASI 90 or PASI 100 experiencing the greatest quality of life benefits [41]. The finding that risankizumab was associated with the lowest absolute DLQI score at 12 months in our cohort is also consistent with the generally better skin clearance rates reported with IL-23 inhibitors in both registry and clinical trial data, and supports the notion that a deeper clinical response directly translates into greater patient-reported improvement in quality of life.

### 4.3. Safety

Biologic therapy is widely used for chronic psoriasis because it can rapidly control the disease and improve quality of life. As a result, its safety profile is an important consideration in routine clinical practice. IL-17 inhibitors are not consistently associated with increased serious infection risk in meta-analyses, but they are associated with increased mucocutaneous candidiasis [43,44]. In head-to-head data, secukinumab was associated with higher rates of candidiasis and inflammatory bowel disease compared to ustekinumab (2.4% vs. 0.7% and 0.4% vs. 0%, respectively), although these events were consistent with previously reported secukinumab safety findings and no new safety signals were identified, including malignancy, tuberculosis reactivation, or opportunistic infections [25,45,46]. In contrast, TNF-α inhibitors have established safety concerns, including serious infections, reactivation of tuberculosis or hepatitis B, demyelinating disease, congestive heart failure, malignancy, and paradoxical psoriasis, with guideline recommendations emphasising screening and risk-based avoidance in specific populations [33,47,48,49,50,51]. Registry and observational studies suggest that prolonged exposure to TNF-α inhibitors may be associated with an increased risk of malignancy. In addition, cohorts of patients with inflammatory dermatoses treated with targeted therapies have reported quantified rates of skin cancer [52,53]. Comparative data suggest that IL-23 inhibitors may be associated with fewer overall adverse events than IL-17 inhibitors, possibly because infection rates are higher with IL-17 therapies, while malignancy risk appears low in both groups [54]. Across IL-23 and IL-12/23 agents, the most reported adverse events include nasopharyngitis, upper respiratory tract infection, and injection-site reactions. Network meta-analyses suggest that risankizumab may have a relatively favourable short-term safety profile among IL-23 inhibitors [55,56,57].

In our study, one patient treated with adalimumab developed multiple non-melanoma skin tumours, prompting a switch to ustekinumab; however, we did not observe an overall association between biologic exposure and non-melanoma skin tumours in the cohort. In addition, one woman treated with adalimumab developed pulmonary tuberculosis, highlighting the importance of infection screening and vigilance during TNF-α inhibitor therapy.

### 4.4. Drug Survival

At 1 year, the crude drug survival for effectiveness reported in a real-world cohort was 0.81 for adalimumab (95% CI, 0.80–0.82), 0.89 for ustekinumab (95% CI, 0.88–0.89), 0.86 for secukinumab (95% CI, 0.85–0.87), 0.94 for guselkumab (95% CI, 0.92–0.96) and 0.86 for ixekizumab (95% CI, 0.83–0.89). In multivariable-adjusted models, guselkumab had the highest survival vs. ustekinumab (adjusted HR, 0.13; 95% CI, 0.03–0.56), whereas adalimumab had the lowest survival (adjusted HR, 2.37; 95% CI, 2.03–2.76); secukinumab and ixekizumab showed broadly similar survival patterns over time [58].

Several factors modified effectiveness-related drug survival, including psoriatic arthritis, prior biologic exposure, nail involvement, and ethnicity, with the number of previous biologic therapies emerging as an especially important effect modifier. Among biologic-naive patients, ixekizumab survival was not significantly different from guselkumab (HR, 3.89; 95% CI, 0.73–20.73), and secukinumab showed survival similar to ustekinumab in biologic-naive patients; however, when IL-17 inhibitors were used as third-line or later therapy, attrition was high [58].

These findings are broadly consistent with other registry and cohort studies. Torres et al. reported a 24-month survival function of 0.92 for guselkumab (discontinuations restricted to ineffectiveness) versus 0.84 for ixekizumab and 0.78 for secukinumab [59]. Lockshin et al. reported ixekizumab drug survival of 0.68 (95% CI, 0.54–0.79) at 24 months and a lower discontinuation risk compared with a pooled TNF inhibitor group (HR, 0.36; 95% CI, 0.27–0.47) [60]. In the Austrian Psoriasis Registry, Graier et al. reported 12-month survival of 0.86 (95% CI, 0.82–0.90) with ixekizumab [61].

In our study, drug persistence was generally high across biologics, with a gradual decline over follow-up. At 1 year, drug survival was 75.0% for adalimumab, 82.6% for ustekinumab, 96.0% for guselkumab, 85.7% for secukinumab, 80.0% for etanercept, and 91.7% for infliximab, while risankizumab remained at 100%. By 5 years, survival had fallen to 56.0% (adalimumab), 43.5% (ustekinumab), 88.0% (guselkumab), 95.8% (risankizumab), 33.3% (infliximab), 10.0% (etanercept), and 42.9% (secukinumab). In our cohort, risankizumab appeared to have the highest long-term persistence and etanercept the lowest, though these observations should be interpreted cautiously given the small sample sizes of several subgroups and the exploratory nature of this analysis.

### 4.5. Switching of Biologics

Switching biologic therapy is common in moderate-to-severe psoriasis. As more biologics have become available, switching within or between drug classes has become routine, most often because of insufficient efficacy, adverse events, cost, or reimbursement requirements [62,63,64].

Real-world data from France suggest characteristic switching patterns: patients treated with etanercept most often switch to adalimumab, whereas patients treated with adalimumab commonly switch to ustekinumab or secukinumab. Those switching from etanercept are also more likely to move to another TNF-α inhibitor than those switching from adalimumab, possibly because etanercept is generally considered less effective and switching to adalimumab may represent an escalation within the TNF-α class [65]. Outcomes after switching may also depend on prior exposure: patients switching to ixekizumab from TNF inhibitors, IL-12/23 inhibitors, or IL-23 inhibitors were more likely to achieve PASI 75/90/100 and other clinical targets than those switching from secukinumab [66]. Patient factors can also shape biologic choice; for example, ustekinumab and ixekizumab are recommended in obesity, while TNF inhibitors (especially etanercept) are recommended when chronic infections such as human immunodeficiency virus (HIV) or hepatitis B/C are present [67].

In Lithuania, treatment aims for remission or low disease activity within 3 months; if improvement is not achieved, therapy is adjusted according to PASI and DLQI response thresholds. Treatment response is assessed based on changes in the following indicators: if the PASI score has improved by ≥75%, treatment is continued; if the PASI score has improved by <50%, treatment is changed; if the PASI score has improved by 50–74% and the DLQI score is ≤5, treatment is continued; if the DLQI score is >5, treatment is changed. Treatment with biologic therapy is suspended or discontinued if adverse drug reactions occur. Treatment is initiated with a TNF-α blocker, a PDE4 inhibitor with the lowest cost, or an IL inhibitor (except ustekinumab). However, if the patient also has end-stage heart failure, end-stage renal failure, or demyelinating diseases of the nervous system, treatment is initiated with an IL inhibitor. In patients with suppurative hidradenitis, the first-line drug is adalimumab; in patients with chronic inflammatory bowel disease or chronic inflammatory eye disease, the first-line drug is another generic TNF-α blocker other than etanercept [37].

In our study, during 5 years of follow-up, 53 patients required at least one switch, most often due to insufficient efficacy (93.8%). Switches due to safety concerns were uncommon and included: multiple non-melanoma skin tumours in one adalimumab-treated patient, adalimumab-induced paradoxical psoriasis in another, gastroesophageal reflux disease in one patient, and a case of blurred vision with injection-site pruritus and burning in the anal and urethral regions.

### 4.6. Comorbidities Associated with Psoriasis

Psoriasis is now widely recognised as a chronic systemic inflammatory disorder extending beyond skin and joint involvement, encompassing a broad spectrum of clinically important comorbidities. These include psoriatic arthritis, cardiometabolic diseases (obesity, hypertension, diabetes mellitus, and dyslipidaemia), gastrointestinal disease (particularly inflammatory bowel disease and Crohn’s disease), chronic kidney disease, psychosocial disorders, infections, and malignancies [2].

Hypertension is more common among patients with psoriasis. A meta-analysis of 24 observational studies reported a pooled odds ratio of 1.58 (95% CI, 1.42–1.76) for the association between psoriasis and hypertension, and two cohort studies also found an increased risk of developing new-onset hypertension [68,69]. Hypertension may also be more severe and less well controlled in psoriasis, with poorer control increasing with psoriasis severity independent of BMI and other factors [2,70].

Psoriasis is associated with an increased risk of cardiovascular disease (CVD), likely reflecting both shared risk factors and psoriasis-related systemic inflammation. Patients with psoriasis more often have overweight/obesity, diabetes, hypertension, and a more atherogenic lipid profile [24,71,72]. A large epidemiologic analysis of the United Kingdom General Practice Database reported a higher-than-normal incidence of myocardial infarction among patients with psoriasis [33,73]. Retrospective analyses also suggest that TNF-α inhibitor therapy may improve endothelial function and may be associated with a reduced risk of myocardial infarction [74].

Dyslipidaemia may also be more prevalent in psoriasis. In one systematic review, 20 of 25 studies reported significant associations between psoriasis and dyslipidaemia [75]. Beyond standard lipid measures, advanced lipid testing has demonstrated more atherogenic lipid characteristics and reduced high-density lipoprotein cholesterol efflux capacity in psoriasis patients [2].

Patients with psoriasis have a higher incidence of type 2 diabetes, even after adjustment for major confounders such as BMI, smoking, hypertension, hyperlipidaemia, and cardiovascular disease [76]. Prospective cohort studies suggest that this risk increases in a dose-dependent manner with greater body surface area involvement and may be higher in patients with psoriatic arthritis [77]. Shared genetic background and potential causal mechanisms have also been proposed, including overlap in NF-κB signalling pathways [78]. Moreover, diabetic patients with psoriasis appear to be more likely to require pharmacologic management and to experience microvascular and macrovascular complications than those without psoriasis [2,79,80].

A U.S. study reported significantly higher BMI among psoriasis patients than in the general population [71]. Another research from the U.S. has shown that obesity and weight gain are strong risk factors for the development of psoriasis in women [81]. Multivariate analyses have shown that the risk of developing psoriasis increases with rising BMI, with the highest relative risk observed in individuals in the highest BMI categories, whereas a low BMI (<21) is associated with a reduced risk. Consistent with this association, participants in large biologic psoriasis trials typically have mean body weights of approximately 90–95 kg [31,33,82].

Psoriatic onychodystrophy and psoriatic arthropathy are among the most common and clinically significant comorbidities associated with psoriasis, reflecting the systemic inflammatory nature of the disease [83,84]. The high prevalence of psoriatic onychodystrophy (83.8%) and psoriatic arthropathy (52.9%) observed in our cohort is consistent with the highly selected nature of the study population. Both conditions are well-established markers of psoriasis severity and are consistently more prevalent in biologic-eligible populations than in the general psoriasis population. Published data confirm that nail psoriasis affects 50–79% of patients with skin psoriasis, with rates up to 82% reported across psoriasis cohorts [83,85]. Similarly, the risk of psoriatic arthritis increases markedly with disease severity—reaching an incidence of 17.6 events per 100 patient-years in patients receiving biologics compared with 2.1 in those on topical therapy alone—and population-based Swedish registry data have demonstrated a 3.34-fold higher risk of psoriatic arthritis diagnosis in biologic-treated patients compared with less severely affected groups [85,86]. These findings suggest that the high prevalence figures observed in our cohort may be consistent with the clinical profile expected in a tertiary biologic-treated population, although this interpretation should be considered exploratory given the limitations of the present study.

These comorbidities complicate routine care and should be considered alongside disease severity and patient-specific factors when selecting biologic therapy. Persistent inflammation combined with cardiometabolic and other comorbidities can further impair quality of life and influence treatment choices [23].

In our study, hypertension was the most common comorbidity (17.5%), followed by dyslipidaemia (7.3%) and obesity (7.1%), consistent with prior reports that cardiometabolic conditions are frequent in psoriasis.

### 4.7. Limitations

There are several limitations to our study. First, the sample size was small (*n* = 210), and since adalimumab became the first-line biologic therapy in Lithuania in 2019, most participants have received it. As a result, comparisons of efficacy with infliximab, ustekinumab, and etanercept were limited by the small number of patients treated with these medications. Furthermore, several biologic subgroups had very small sample sizes—most notably secukinumab (*n* = 7) and etanercept (*n* = 10)—which substantially limits the statistical power of between-group comparisons involving these agents; results pertaining to these groups should therefore be interpreted with caution, and no definitive comparative conclusions between biologics should be drawn from these data alone. Second, a dedicated analysis of adolescent patients could not be performed due to the very small number of adolescents included in the cohort (*n* = 2); future studies with larger paediatric populations are needed to evaluate the effectiveness and safety of biological therapy in this age group. Third, the high prevalence of concomitant topical therapies and methotrexate use across all biologic cohorts represents a potential confounding factor, as these co-interventions may have contributed to the observed effectiveness outcomes and should be considered when interpreting the results of the present study. Fourth, data on the involvement of specific difficult-to-treat anatomical sites—including the scalp, genital area, and palmoplantar regions—were not systematically collected as separate variables in our retrospective dataset, as anatomical involvement was recorded according to standard PASI body regions; future prospective studies from our centre will incorporate dedicated assessment of these sites. Fifth, no separate formal statistical comparison of baseline characteristics between biologic treatment groups is presented; however, the key baseline covariates were incorporated into the covariate-adjusted linear mixed-effects model described in Section 3.2.1, which partially accounts for baseline differences between groups. Residual confounding due to unmeasured or incompletely adjusted variables cannot be excluded.

## 5. Conclusions

Biologic therapy was associated with sustained improvement in PASI and DLQI across all treatment classes over five years, with significant between-drug differences in treatment trajectories identified after adjustment for baseline patient characteristics. Etanercept demonstrated the lowest long-term drug survival, while switching due to insufficient efficacy frequently restored clinically meaningful responses in subsequent lines. These findings provide real-world evidence of biologic efficacy and drug survival from Lithuania, contributing data from a Baltic region setting currently underrepresented in the international literature.

## Figures and Tables

**Table 1:** Baseline demographic and clinical characteristics.

Baseline Demographic and Clinical Characteristics	Adalimumab (*n* = 84)	Etanercept (*n* = 10)	Infliximab (*n* = 12)	Risankizumab (*n* = 24)	Guselkumab (*n* = 25)	Secukinumab (*n* = 7)	Ustekinumab (*n* = 46)	Total (*n* = 208)
**General Demographics**								
Males, % (*n*)	56.0 (47)	60.0 (6)	83.3 (10)	70.8 (17)	72.0 (18)	28.6 (2)	54.3 (25)	60.1 (125)
Females, % (*n*)	44.0 (37)	40.0 (4)	16.7 (2)	29.2 (7)	28.0 (7)	71.4 (5)	45.7 (21)	39.9 (83)
Age at enrolment, mean, years (SD)	48.0 (13.4)	47.9 (13.7)	48.1 (13.7)	48.1 (13.4)	48.3 (13.6)	45.0 (12.5)	48.0 (13.4)	48.3 (13.5)
Positive family history of psoriasis, % (*n*)	33.3 (28)	30.0 (3)	25.0 (3)	20.8 (5)	24.0 (6)	14.3 (1)	30.4 (14)	28.6 (60)
Negative family history of psoriasis, % (*n*)	14.3 (12)	30.0 (3)	0.0 (0)	8.3 (2)	8.0 (2)	14.3 (1)	13.0 (6)	12.4 (26)
No data of family history of psoriasis, % (*n*)	52.4 (44)	40.0 (4)	75.0 (9)	70.8 (17)	68.0 (17)	71.4 (5)	56.5 (26)	58.7 (122)
**Disease Characteristics**								
Mean disease duration, years (SD)	24.2 (11.8)	24.4 (12.1)	24.6 (12.1)	24.1 (11.8)	24.5 (12.0)	23.1 (11.0)	24.2 (11.9)	24.5 (12.0)
Baseline BMI, kg/m^2^ (SD)	28.1 (5.2)	27.6 (4.3)	33.6 (11.2)	28.9 (7.9)	31.6 (7.0)	32.6 (9.8)	29.6 (8.4)	30.0 (7.0)
Psoriatic arthropathy, % (*n*)	48.8 (41)	70.0 (7)	91.7 (11)	29.2 (7)	56.0 (14)	71.4 (5)	52.2 (24)	52.4 (109)
Psoriatic onychodystrophy, % (*n*)	79.8 (67)	100.0 (10)	91.7 (11)	66.7 (16)	92.0 (23)	100.0 (7)	87.0 (40)	83.7 (174)
**Biologic Treatment History**								
Biologic-naïve, % (*n*)	91.7 (77)	100.0 (10)	100.0 (12)	87.5 (21)	80.0 (20)	71.4 (5)	80.4 (37)	80.8 (162)
Biologic-experienced, % (*n*)	8.3 (7)	0.0 (0)	0.0 (0)	12.5 (3)	20.0 (5)	28.6 (2)	19.6 (9)	19.2 (46)S

Abbreviations: BMI, body mass index; SD, standard deviation.

**Table 2:** Distribution of concomitant topical and systemic treatments across biologic cohorts.

Concomitant Treatment	Adalimumab (*n* = 84)	Ustekinumab (*n* = 46)	Guselkumab (*n* = 25)	Risankizumab (*n* = 24)	Infliximab (*n* = 12)	Etanercept (*n* = 10)	Secukinumab (*n* = 7)
Topical emollients, % (*n*)	98.8 (83)	97.8 (45)	100.0 (25)	100.0 (24)	100.0 (12)	100.0 (10)	100.0 (7)
Topical corticosteroids, % (*n*)	97.6 (82)	95.7 (44)	92.0 (23)	87.5 (21)	100 (12)	90.0 (9)	85.7 (6)
Keratolytic agents, % (*n*)	53.6 (45)	56.5 (26)	36.0 (9)	25.0 (6)	50.0 (6)	60.0 (6)	57.1 (4)
Topical calcineurin inhibitors, % (*n*)	8.3 (7)	4.4 (2)	36.0 (9)	12.5 (3)	0 (0)	10.0 (1)	14.3 (1)
Topical corticosteroids + vitamin D analogues, % (*n*)	38.1 (32)	26.1 (12)	28.0 (7)	37.5 (9)	25.0 (3)	20.0 (2)	14.3 (1)
Vitamin D analogues, % (*n*)	2.4 (2)	4.4 (2)	4.0 (1)	4.2 (1)	8.3 (1)	10.0 (1)	0 (0)
Concomitant methotrexate during biologic therapy, % (*n*)	35.7 (30)	17.4 (8)	20.0 (5)	20.8 (5)	83.3 (10)	20.0 (2)	42.9 (3)

**Table 3:** Dermatological and non-dermatological comorbidities.

Comorbidity	*n* (%)
**Dermatological** **Comorbidities**	169 (32.3)
**Inflammatory skin diseases**	
Seborrheic dermatitis	16 (9.5)
Rosacea	12 (7.1)
Acne vulgaris	14 (8.3)
Hidradenitis suppurativa	6 (3.6)
Other inflammatory skin diseases *	9 (5.3)
**Other dermatological conditions**	112 (21.4)
**Non-Dermatological Comorbidities**	354 (67.7)
**Cardiometabolic & cardiovascular**	
Hypertension	62 (17.5)
Dyslipidaemia	26 (7.3)
Obesity	25 (7.1)
Diabetes mellitus, type 2	10 (2.8)
Other cardiometabolic & cardiovascular **	22 (6.2)
**Infectious diseases**	
Coronavirus disease (COVID-19)	17 (4.8)
Acute upper respiratory tract infection	13 (3.7)
Latent tuberculosis infection	10 (2.8)
Urinary tract infection	9 (2.5)
Pneumonia	4 (1.1)
Other infectious diseases ***	17 (4.8)
**Other non-dermatological conditions**	143 (27.3)
**In total**	523 (100)

* Includes dermatitis 3 (1.8), urticaria 2 (1.2), metal-induced allergic contact dermatitis 2 (1.2), atopic dermatitis 1 (0.6), herpes zoster 1 (0.6). ** Includes fatty liver disease 15 (4.2), cardiomegaly 4 (1.1), aortic atherosclerosis 2 (0.6), myocardial infarction 1 (0.3). *** Includes subacute and chronic vaginitis 6 (1.7), chronic bronchitis 3 (0.9), tonsillitis 2 (0.6), hepatitis B 2 (0.6), acute bronchitis 2 (0.6), urethritis 4 (1.1), pulmonary tuberculosis 1 (0.3), hepatitis C virus 1 (0.3), Lyme disease 1 (0.3), Clostridium difficile enterocolitis 1 (0.3).

**Table 4:** Unadjusted hazard ratios for the difference in drug survival, based on Cox proportional hazards regression models.

Drug Group	HR	95% CI	*p* Value
Etanercept vs. ustekinumab	2.55	1.17–5.52	0.018
Infliximab vs. etanercept	0.36	0.13–0.97	0.043
Adalimumab vs. etanercept	0.47	0.23–0.98	0.045
Adalimumab vs. ustekinumab	0.94	0.58–1.53	0.795
Adalimumab vs. infliximab	0.93	0.44–1.98	0.854
Infliximab vs. ustekinumab	0.97	0.44–2.16	0.949

## Data Availability

The data supporting the findings of this study are available from the corresponding author upon reasonable request.

## Abbreviations

The following abbreviations are used in this manuscript:

PASI	Psoriasis Area and Severity Index
DLQI	Dermatological quality of life index
HR	Hazard ratio
CI	Confidence interval
PASI 50	≥50% reduction from the baseline in PASI scores
PASI 75	≥75% reduction from the baseline in PASI scores
PASI 90	≥90% reduction from the baseline in PASI scores
PASI 100	≥100% reduction from the baseline in PASI scores
TNFi	Tumour necrosis factor inhibitor
IL-17i	Interleukin 17 inhibitor
IL-12/23i	Interleukin 12/23 inhibitor
IL-23i	Interleukin 23 inhibitor
PsA	Psoriatic arthritis
VUH SK DVC	Vilnius University Hospital Santaros Klinikos, Centre of Dermatovenereology
BMI	Body mass index
NA	Not applicable
SD	Standard deviation
REML	Restricted maximum likelihood
UVB	Ultraviolet B phototherapy
HIV	Human immunodeficiency virus
US	United States
COVID-19	Coronavirus disease
